# Analysis of TGFβ1-Induced activin A gene expression in kidney mesangial cells

**DOI:** 10.3389/fmolb.2025.1607043

**Published:** 2025-09-23

**Authors:** Asfia Soomro, Ifeanyi Kennedy Nmecha, Jackie Trink, Renzhong Li, Joan C. Krepinsky

**Affiliations:** Division of Nephrology, Department of Medicine, McMaster University, Hamilton, Canada

**Keywords:** activin A, TGFβ1, kidney fibrosis, promoter activity, regulatory elements

## Abstract

**Introduction:**

The cytokine activin A is emerging as an important regulator of kidney fibrosis. Its expression, negligible in normal kidney, is significantly increased in various fibrotic kidney diseases. TGFβ1 is a cytokine belonging to the same family, which is well established to be a central mediator of kidney fibrosis. Although targeting TGFβ1 therapeutically is not feasible due to its homeostatic roles, we previously showed that activin A is upregulated by, and mediates the profibrotic effects of, TGFβ1.

**Methods:**

We investigated the transcriptional regulation of activin A by TGFβ1 in primary kidney mesangial cells (MC). Cells were transfected with a luciferase reporter construct containing the activin A promoter or a series of deletion constructs. Guided by MatInspector, key TGFβ1-responsive consensus elements were identified.

**Results:**

TGFβ1 increased transcription of the activin A subunit *inhba*. Using a series of deletion constructs of the *inhba* promoter, we identified a critical regulatory region located 350bp from the transcription start site that is responsive to TGFβ1. Analysis of this region for transcription factor regulatory elements, coupled with mutation analyses and transcription factor downregulation with siRNA, showed that Stat5 and FoxP1, but not Sox9, regulate *inhba* transcription by TGFβ1. Interestingly, although no consensus binding site in this region was identified for Smad3, a well-established mediator of TGFβ1 signaling, both a Smad3 inhibitor and use of MC isolated from Smad3 knockout kidneys, showed its requirement for the TGFβ1 response. We further identified a CT microsatellite just upstream of 350bp which suppressed promoter activity.

**Conclusion:**

These findings provide insight into potential therapeutic targets for activin A targeting and attenuation of kidney fibrosis.

## 1 Introduction

Chronic kidney disease is a major public health issue, estimated to affect 10%–13% of the global population (Kovesdy, 2022). Regardless of cause, it is marked pathologically by progressive fibrosis that often leads to kidney failure requiring dialysis or transplantation to sustain life (Ruiz-Ortega et al., 2020). The cytokine transforming growth factor-beta 1 (TGFβ1) has been recognized as a key mediator of kidney fibrosis, but its targeting is not feasible due to its multifaceted role in homeostasis (Trionfini and Benigni, 2017). We recently identified an important role for the TGFβ family member activin A in mediating the longer-term profibrotic effects of TGFβ1 (Soomro et al., 2023). Not expressed in normal kidney, activin A is significantly upregulated in kidney fibrosis in rodent models and in humans, and by TGFβ1 in kidney cells (Zhang et al., 2019; Soomro et al., 2023; Tsai et al., 2023). Circulating and urinary activin A have also been correlated with progressive kidney disease (Bian et al., 2019; Lima et al., 2019; Tsai et al., 2023). Understanding the mechanism by which activin A is upregulated may thus provide a potential avenue to inhibit its increased expression in disease to attenuate fibrosis.

Activin A is synthesized as a homodimer of inhibin βA subunits. Each subunit is formed as a proprotein containing an N-terminal prodomain and a C-terminal mature domain. The prodomain is cleaved by proprotein convertases, remaining noncovalently associated with the disulfide-linked homodimer of the C-terminal mature region and active protein (Zhang et al., 2017). It can be displaced by high affinity interaction of the mature activin with its type IIA or B transmembrane receptor. Recruitment of the type I receptor Alk4 or Alk7 to the complex induces biological effects through Smad and non-Smad signaling pathways (Namwanje and Brown, 2016).

The inhibin βA (*inhba*) gene, encoding for the activin A subunit, exhibits >97% conservation across species (Hedger and de Kretser, 2013; Billings et al., 2020). Its regulation has been studied in various cell types including monocytes/macrophages, placental cells, bone marrow stromal cells, and epithelial cells in testis and ovary. These studies show that *inhba* gene expression can be induced by several signaling pathways involved in inflammation and immune regulation requiring AP-1, CREB, c-MAF and NFAT transcription factors (Tanimoto et al., 1996; Ardekani et al., 1998; Ogawa et al., 2006). To date, the means by which TGFβ1 induces *inhba* synthesis is as yet unknown. In the present study, we have investigated the regulation of *inhba* transcription by TGFβ1 in kidney glomerular mesangial cells (MC) using a series of promoter deletion and mutation constructs to identify important regulatory elements. These studies lay the foundation for development of therapeutics which may indirectly target TGFβ1 profibrotic effects by reducing *inhba* and activin A expression.

## 2 Methods

### 2.1 Cell culture

Primary mouse MC cultures were previously established. MC were outgrown from glomeruli of male C57BL/6 wild-type or Smad3 knockout mice isolated using Dynabeads (Invitrogen). Cells were cultured in Dulbecco’s modified eagle’s medium supplemented with 16% fetal bovine serum, streptomycin (100 μg/mL) and penicillin (100 μg/mL) at 37 °C in 95% O_2_, 5% CO_2._ Cells from passages 10 to 13 were used for transfection experiments. They were serum deprived at 80%–90% confluence in 1% bovine serum albumin (BSA) for 24 h following transfection and prior to treatment with any of 0.5 ng/mL TGFβ1 (R&D Systems), 94 mM Stat5 inhibitor (Cayman Chemical), 1 µg actinomycin D (Thermo Fisher) or 5uM Smad3 inhibitor SIS3 (Cayman Chemical).

### 2.2 Plasmids

DNAzol (Invitrogen) was used to isolate genomic DNA from mouse MC. The promoter region 2048bp upstream of the start codon for *inhba* (NCBI Reference Sequence: XM_011244285.3) was amplified using the following primers with *MluI* and *XhoI* restriction enzyme sites attached to the 5′ and 3′ end respectively: F5′-ACTACGCGTAAAACTGAAGTTTAACCTAGTGTC-3′, R5′- CCTGGCAGCAAAAGTCGTG -3’. The following forward primers were used to create deletion constructs in conjunction with the above reverse primer: 686bp-actA F5′-ACTACGCGTGAGCAAGGAGCAGCAAGAA-3′, 647bp-actA F5′-ACTACGCGTCCCTAGTCACAGCTCATACT-3′, 577bp-actA F5′-ACTACGCGTAATCCCTCTCTCTCTCTCTC-3′, 437bp-actA F5′-ACTACGCGTCTCTCTTCCTTTCTCCCTCC-3′, 350bp-actA F5′-ACTACGCGTCTGTCTCTCCCTCCCATC-3′, 248bp-actA F5′-ACTACGCGTCTCCCTCTCTCCCTCCCTCC-3′, 175-actA F5′-ACTACGCGTTGCATTCAGAGAGGGAACC -3’. PCR products were purified after gel electrophoresis and ligated into the pGL3 firefly luciferase plasmid (Promega) using appropriate restriction enzymes. All plasmids were sequenced.

Using the −350bp plasmid as a template, one predicted FoxP1 (AAACA) and one Stat5 (TTCACAGA) binding site were identified using MatInspector (Genomatrix, Germany). Each site was mutated separately to generate the 350bp ActA-FoxP1-mut and 350bp ActA-Stat5-mut*,* using two sets of primers, P1 and P2, respectively. Briefly, each construct was created using P1 and P2 to mutate the binding site. Two parts of each construct were created to mutate the region where the binding site for FoxP1 or Stat5 is located. An additional PCR was completed to create one final construct containing the full −350bp sequence containing the mutated site. These mutated −350bp constructs were then cloned into the high efficiency TOPO TA cloning vector. Established clones were selected, purified DNA was digested and ligated into a pGL3 luciferase vector. The following primers were used for site-directed mutagenesis: P1 350bp-FoxP1-mut; sense, F5′-ACTACGCGTCTGTCTCTCCCTCCCATC-3’; antisense, R5′-CTCTCCCTTGGACGAAACAT-3’. P2 350bp-FoxP1-mut; sense, F5′- GAGAGGGAACCTGCAAACAA-3’; antisense, R5′- CCTGGCAGCAAAAGTCGTG -3’. P1 350bp-Stat5-mut; sense, F5′-ACTACGCGTCTGTCTCTCCCTCCCATC-3’; antisense, R5′- TGTTTTGAAGTGTCTTTTG -3’. P2 350bp-Stat5-mut; sense, F5′- ACAAAACTAGTGTCTTAAC -3’; antisense, R5′- CCTGGCAGCAAAAGTCGTG -3’.

The pcDNA3.1 FoxP1A plasmid was a gift from Dr. A. Rao (Addgene #16362).

### 2.3 Luciferase and transient transfections

For luciferase experiments, 3 × 10^5^ MC were plated in 12-well plates in triplicate at 60%–70% confluence and transfected with 0.5 μg of promoter luciferase plasmid along with 0.05 μg pCMV β-galactosidase (Clontech) using Effectene (Qiagen). After the cells were harvested, luciferase and β-galactosidase activities were measured using kits (Promega) and a SpectraMax L Microplate Reader (Molecular Devices) set to measure luminescence or 420 nm absorbance respectively.

For siRNA transfection, 4 × 10^5^ MC were seeded in a 6-well plate and allowed to reach a confluence of 30%–40%. Knockdown of Sox9 and FoxP1 was achieved using 50 nM specific siRNA (Thermo Fisher) with RNAiMAX (Thermo Fisher).

### 2.4 PCR

TRIzol Reagent (Invitrogen) was used for RNA extraction. For all samples, 1 µg RNA was reverse transcribed to cDNA using qScript cDNA SuperMix Reagent (Quanta Biosciences) for quantitative real-time PCR using Power SYBR Green PCR Master Mix (Thermo Fisher Scientific). Specific primers used were: *Inhba* F5′-ACAGCCAGGAAGACACTGCA-3′ and R5′-CAGGTCACTGCCTTCCTTGG-3’; 18S F5′-GCCGCTAGAGGTGAAATTCTTG-3′, R5′-CATTCTTGGCAAATGCTTTCG-3′ as internal control. Gene expression was calculated using the ∆∆C_T_ method.

### 2.5 Protein extraction and immunoblotting or ELISA

Protein was extracted using lysis buffer (20 mM Tris-HCl pH 7.5, 150 mM NaCl, 1% Triton X-100, 1 mM EDTA, 1 mM EGTA, 2.5 mM sodium pyrophosphate, 1 mM β-glycerophosphate, 2 mM DTT, 1 mM sodium vanadate, 1 mM phenylmethylsulphonyl fluoride, 1 μg/mL leupeptin, 2 μg/mL aprotinin). Cell lysates were then centrifuged and the resulting supernatant used to measure activin A (R&D Systems), Smad3 (Abcam), FoxP1 (Cell Signaling), Stat5 (R&D Systems), Sox9 (R&D Systems), tubulin (Sigma) and GAPDH (Millipore) levels by immunoblotting or with the activin A Quantikine ELISA Kit (R&D Systems).

### 2.6 Nuclear extraction and immunoprecipitation

As described previously (Uttarwar et al., 2012), cells were lysed using hypotonic buffer for nuclear protein purification. Once centrifuged, pelleted nuclei were sonicated with hypotonic buffer composed of 0.4M NaCl and 10% glycerol. Equal amounts of nuclear protein were immunoprecipitated using 1 µg Smad3 antibody overnight at 4 °C, followed by incubation with protein G-agarose slurry for 2 h (4 °C). After washing, total immunoprecipitated products were separated on SDS-PAGE for immunoblotting.

### 2.7 Chromatin immunoprecipitation (ChIP)

This was conducted as previously described (Mehta et al., 2019). Briefly, after MC were treated with TGFβ1 for 6 h, protein-DNA complexes were crosslinked using formaldehyde, nuclear extracts were sheared by sonication followed by immunoprecipitation with FoxP1, Stat5 or nonspecific IgG antibodies (1 µg). After reversing crosslinking, DNA was isolated and used for qRT-PCR using primers for the −350bp promoter region. 10% of the original nuclear isolate before immunoprecipitation was used as input. Ct values were evaluated across 3 separate experiments, with target enrichment of Stat5 or FoxP1 calculated by normalizing to % input.

### 2.8 *In vivo* validation using immunofluorescence

Animal studies were previously carried out in accordance with the principles of laboratory animal care and McMaster University and Canadian Council on Animal Care guidelines (protocol #22–05–17). Male C57BL/6 mice underwent resection of the upper and lower poles of the left kidney at 6–7 weeks of age, followed by right nephrectomy after a 1-week recovery period. Kidneys were harvested after 16 weeks and stored in OCT compound. For immunofluorescence, 8 µm sections were cut, fixed in 3.7% paraformaldehyde, permeabilized with 0.2% Triton X-100 and blocked prior to overnight incubation at 4 °C with a primary antibody against activin A (1:200, R&D Systems). The following day, primary antibodies against FoxP1 (1:50) or Stat5 (1:1000) were applied overnight at 4 °C, followed the next day by incubation with secondary antibodies (Alexa Fluor 488-conjugated anti-goat and Alexa Fluor 568-conjugated anti-rabbit from Invitrogen) for 30 min at room temperature. In a second 5/6 nephrectomy model, male CD-1 mice were used as they display greater injury than the C57BL/6 strain (protocol #23–40). Kidneys in this model were harvested 9 weeks after 5/6 nephrectomy and sections used for costaining with the MC marker integrin α8 (1:250, R&D Systems). Images were acquired using a Nikon Eclipse T*i*2 microscope at ×20 magnification.

### 2.9 Statistical analysis

Values are presented as mean ± SEM. Statistical difference among multiple groups was determined using ANOVA with a Tukey’s post hoc test. Unpaired, two-tailed Student *t* tests were used for single comparisons. P value <0.05 was considered statistically significant, using GraphPad Prism 7 for calculations.

## 3 Results

### 3.1 *Inhba* is increased in kidney disease and by TGFβ1

**FIGURE 1:**
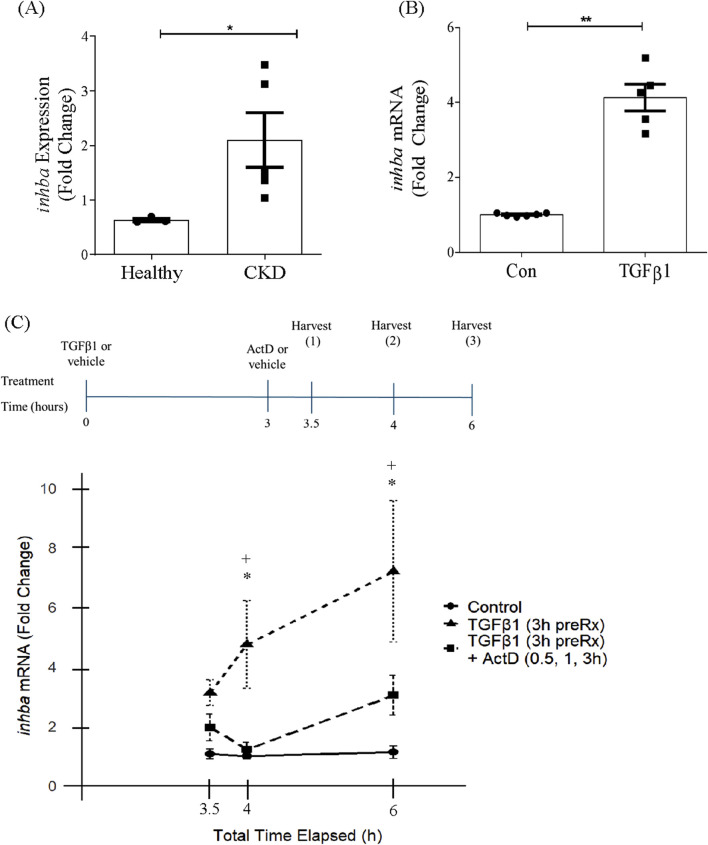
Activin A subunit gene expression is increased in chronic kidney disease and by TGFβ1 in kidney mesangial cells. **(A)** *Inhba* gene is increased in chronic kidney disease (CKD) (n = 5), compared with normal human kidney samples (n = 3), using data available for the Nakagawa study (GSE66494) in the Nephroseq database (www.nephroseq.org). *P < 0.05. **(B)** TGFβ1 increases *inhba* transcript at 24 h (n = 5). **P < 0.01. **(C)** MC were pretreated for 3 h with TGFβ1 to induce expression of *inhba*, followed by addition of vehicle or actinomycin D (ActD) and harvested after a further 30 min, 1 h or 3 h. The treatment timeline is shown above the graph. ActD prevented further synthesis of *inhba*, supporting induction by TGFβ1 at the transcriptional level (n = 5–9) *P < 0.05 TGFβ1 vs. con, ^+^ *P* < 0.05 TGFβ1 vs. TGFβ1+ActD.

We and others have shown increased expression of activin A in both rodent and human chronic kidney disease (Zhang et al., 2019; Soomro et al., 2023; Tsai et al., 2023). To identify whether increased *inhba* transcript is also seen, we accessed the Nephroseq database (www.nephroseq.org) and extracted gene expression information for *inhba* from the Nakagawa study (GSE66494) which included pathologist-confirmed samples for chronic kidney disease (n = 5) and normal (n = 3) human kidney samples. These data show that *inhba* gene expression is significantly increased in chronic kidney disease (Figure 1A).

We have previously shown that TGFβ1 increases synthesis and secretion of activin A in MC (Soomro et al., 2023). To determine whether this was dependent on induction of *inhba* gene expression, MC were treated for 24 h with TGFβ1 and *inhba* assessed by qRT-PCR. Figure 1B shows a significant increase in transcript levels. We next assessed response to TGFβ1 in the presence of actinomycin D, an inhibitor of transcription, to determine whether RNA synthesis was required. MCs were treated with TGFβ1 for 3 h before the addition of actinomycin D and transcript levels were then assessed at 0.5, 1 and 3 h following treatment, as show in Figure 1C. As shown in the graph, TGFβ1 induced ongoing synthesis of *inhba* which was prevented by inhibition of transcription. These data show a direct transcriptional effect of TGFβ1 on *inhba*.

### 3.2 TGFβ1 induction of *inhba* requires elements near the transcription start site

**FIGURE 2:**
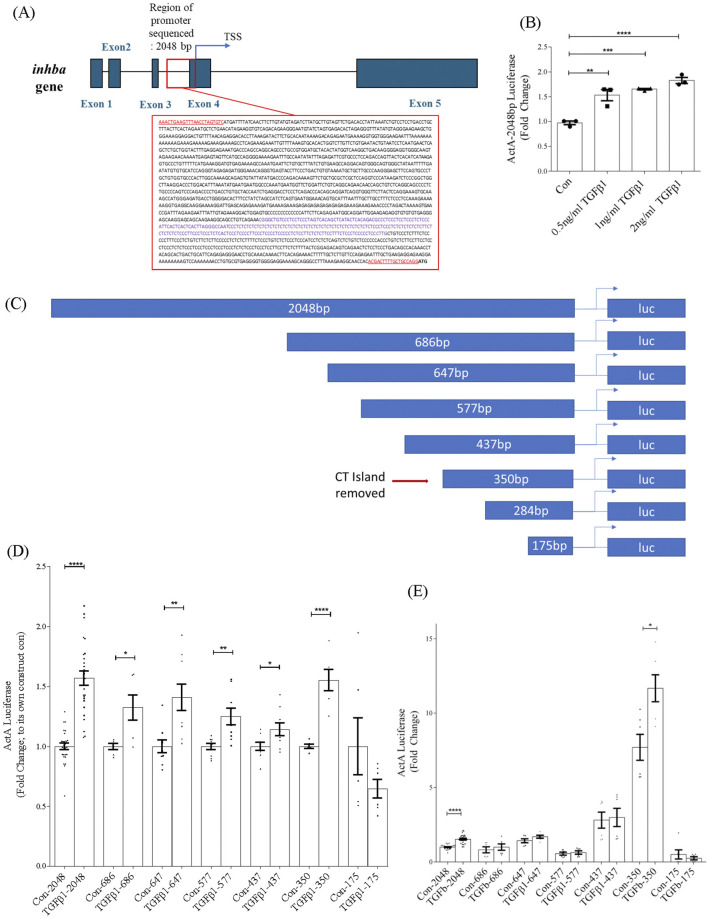
Sequence of *inhba* and its promoter region and identification of the TGFβ1-responsive region of the *inhba* promoter. **(A)** Genetic map of the mouse *inhba* gene showing introns and exons before splicing (MGI:96570, https://www.ncbi.nlm.nih.gov/gene/16323). The promoter region is detailed in the red box. Underlined sequences represent the primers used for amplification. The purple text within the sequence identifies the CT microsatellite. **(B)** Activity of the 2048bp promoter luciferase construct is dose-dependently increased by TGFβ1 (24 h) (n = 3). Subsequent studies used 0.5 ng/mL of TGFβ1. **(C)** Schematic depicting the serial deletion constructs and location of the CT microsatellite region (repetitive dinucleotides of C, cytosine and T, thymine). **(D)** Promoter activation by TGFβ1 for the various deletion constructs shows responsiveness to TGFβ1 in all constructs except for the shortest (175bp), and increased activity with removal of the microsatellite region (n = 6–21). For each construct, data are normalized to their own control. **(E)** Data from panel C are presented relative to the −2048bp control values, highlighting that removal of the CT island significantly increases basal promoter activity. *,**,***,****P < 0.05, 0.01, 0.001, 0.0001 respectively.

To understand how TGFβ1 regulates *inhba* transcription, we cloned 2048 base pairs of the *inhba* promoter upstream of its transcription start site (TSS) (NCBI gene ID 16323) and inserted it upstream of the luciferase gene. This is depicted in Figure 2A which also shows the *inhba* gene structure. We first confirmed activation of the promoter luciferase reporter by TGFβ1 (Figure 2B). To identify regions necessary for the TGFβ1 response, we then created a series of deletion constructs as shown in Figure 2C. These were tested with TGFβ1 0.5 ng/mL. As seen in Figure 2D, the full promoter as well as subsequent deletion constructs were all responsive to TGFβ1, with the exception of the shortest sequence (−175bp). These findings indicate that important TGFβ1-regulatory elements reside in the −350 to −175 region of the *inhba* promoter.

### 3.3 *Inhba* promoter activity is regulated by a CT island microsatellite region

Interestingly, the −350bp construct showed a more robust response to TGFβ1 compared to the −437bp construct. Examination of this region identified a microsatellite region containing a high degree of repetition of the dinucleotides cytosine and thymidine, shown in purple in the boxed region in Figure 2A. Microsatellite regions control the degree of transcriptional activity depending on their location relative to the TSS, causing an expansion or contraction of the length of that region to form an open or closed secondary configuration, respectively (Bagshaw, 2017). The effect of the microsatellite region on overall promoter activity is highlighted in Figure 2E, in which the promoter activity data shown in Figure 2D are presented relative to the −2048bp basal promoter activity rather than to their own internal control. Here, removal of the CT island in the −350bp construct significantly increased basal activity of the promoter. These data imply that the microsatellite region has an overall silencing effect on *inhba* promoter activity.

### 3.4 TGFβ1-induced *inhba* promoter activity requires FoxP1 and Stat5, but not Sox9

**FIGURE 3:**
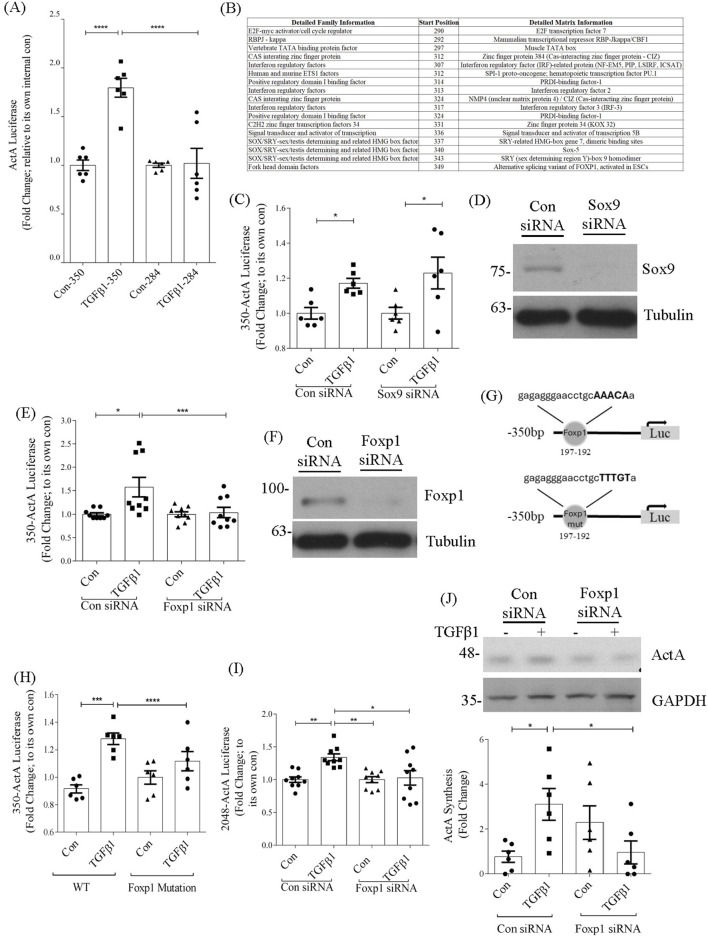
FoxP1, but not Sox9, regulates activation of the *inhba* promoter by TGFβ1. **(A)** TGFβ1 (24 h)-induced promoter activation is no longer seen in the 284bp promoter luciferase construct (n = 5). **(B)** Table displaying a list of possible regulatory elements generated by MatInspector. **(C)** Sox9 knockdown does not inhibit TGFβ1-induced activation of the 350bp promoter (n = 6). **(D)** Sox9 knockdown is confirmed by immunoblotting (n = 2). **(E)** FoxP1 knockdown prevents TGFβ1-induced activation of the 350bp promoter (n = 9–12). **(F)** FoxP1 knockdown is confirmed by immunoblotting (n = 2). **(G)** Schematic showing site-directed mutagenesis of the FoxP1 binding site in the *inhba* promoter, which **(H)** prevents TGFβ1-induced promoter activation (n = 6). **(I)** FoxP1 siRNA also prevents activation of the full length promoter by TGFβ1 (n = 8), as well as production of activin A protein **(J)** (n = 6). *,**,***,****P < 0.05, 0.01, 0.001, 0.0001 respectively.

To identify the regulatory elements within the −350 to −175bp region specific to TGFβ1 induction of *inhba*, a smaller deletion construct was created (−284bp). Figure 3A shows the absence of induction by TGFβ1 for this construct and indicates that important TGFβ1-responsive elements reside in the −350 to −284bp region. Analysis of this region using MatInspector identified several potential regulators as shown in Figure 3B. We chose three transcription factors for further analysis based on their previously identified role as mediators of TGFβ1 profibrotic effects. These include Sex-determining region Y-box gene 9 (Sox9), forkhead box transcription factor 1 (FoxP1) and signal transducer and activator of transcription 5 (Stat5) (Bot et al., 2011; Li et al., 2018; Massague and Sheppard, 2023). Further studies used the −350bp promoter construct.

Using siRNA downregulation, we first assessed whether Sox9 was required for *inhba* upregulation by TGFβ1. As shown in Figure 3C, this did not reduce TGFβ1-induced *inhba* promoter activation, with confirmation of Sox9 downregulation shown in Figure 3D. However, siRNA downregulation of FoxP1 abrogated induction of promoter activity by TGFβ1 (Figure 3E), with its downregulation also confirmed in Figure 3F. Mutation of the FoxP1 regulatory element (Figure 3G) similarly prevented promoter induction by TGFβ1 (Figure 3H). The importance of FoxP1 for regulation of the full promoter construct, as well as synthesis of activin A, was confirmed in Figures 3I,J respectively. Fold changes were calculated by normalizing the measured values of the treatment to the corresponding controls within each siRNA group.

**FIGURE 4:**
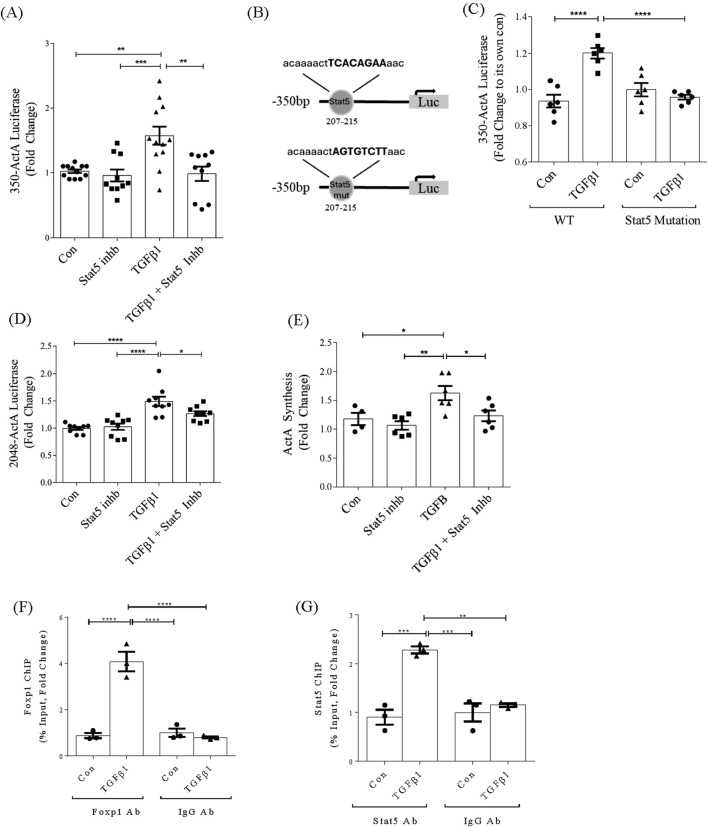
Stat5 and FoxP1 contribute to the induction of *inhba* by TGFβ1 and directly interact with −350bp promoter. **(A)** Stat5 inhibition prevents TGFβ1-induced activation of the 350bp promoter (n = 9). **(B)** Schematic showing site-directed mutagenesis of the Stat5 binding site in the *inhba* promoter. **(C)** Mutation of the Stat5 binding site prevents TGFβ1-induced activation of the 350bp promoter (n = 6). **(D)** Stat5 inhibition prevents TGFβ1-induced activation of the full length promoter (n = 9) and **(E)** activin A synthesis assessed by ELISA of cell lysate (n = 4–6). **(F)** ChIP assays of FoxP1 and **(G)** Stat5 show increased transcription factor association with the −350bp *inhba* promoter region upon TGFβ1 treatment (6 h, n = 3). *,**,***,****P < 0.05, 0.01, 0.001, 0.0001 respectively.

We next assessed whether Stat5 was required for promoter activation. Figure 4A shows that a Stat5 inhibitor prevented TGFβ1-induced *inhba* promoter activation, as did mutation of the Stat5 binding element (Figures 4B,C). Stat5 inhibition also attenuated TGFβ1-induced activation of the full promoter, although not as effectively as for the shorter promoter (Figure 4D). However, a significant reduction in TGFβ1-induced activin A protein production as assessed by ELISA was seen with Stat5 inhibition (Figure 4E).

To confirm binding of FoxP1 and Stat5 to the −350bp promoter region, we performed ChIP assays. Figures 4F,G show binding of both of these transcription factors to this promoter region, with no binding seen when using control IgG for immunoprecipitation from nuclear lysates. These data overall identify an important role for FoxP1 and Stat5, but not Sox9, in regulation of *inhba* by TGFβ1.

### 3.5 Smad3 is required for induction of *inhba* by TGFβ1 in mesangial cells

**FIGURE 5:**
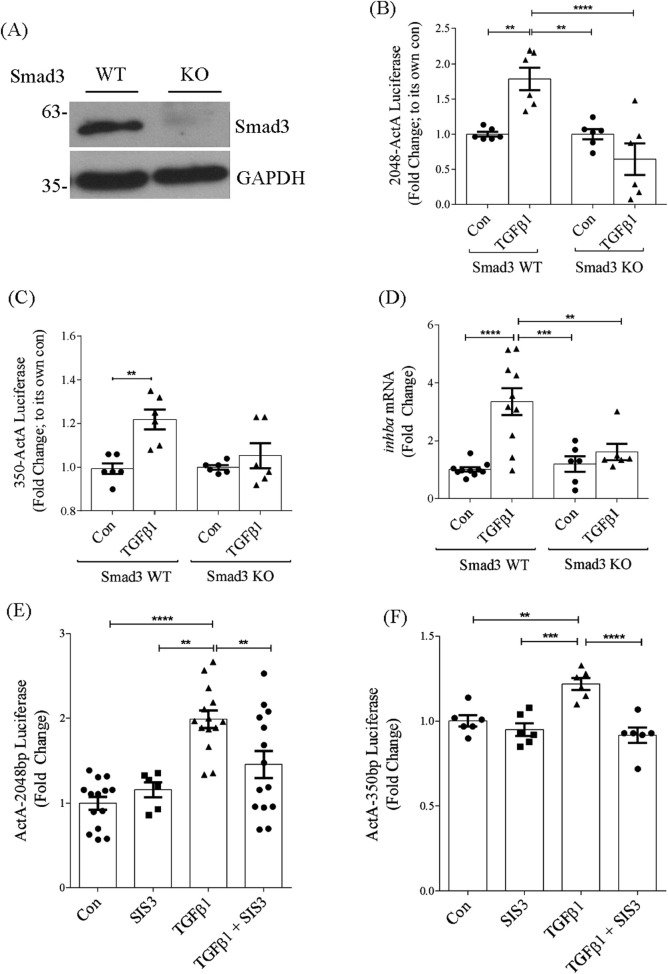
Smad3 is required for TGFβ1-induced *inhba* promoter activation. **(A)** Immunoblot of Smad3 in wild-type and knockout cells showing its absence in Smad3 knockout MC. **(B,C)** TGFβ1 does not increase activation of the full-length **(B)** (n = 6) or 350bp **(C)** (n = 6) promoter in Smad3 knockout MC in comparison to Smad3 wild-type MC. **(D)** Similarly, knockout cells did not show increase in *inhba* transcript in response to TGFβ1 (n = 6). **(E,F)** Smad3 inhibitor SIS3 also decreases TGFβ1-induced activation of the full-length **(E)** (n = 6) or 350bp **(F)** (n = 6) promoter. **,***,****P < 0.01, 0.001, 0.0001 respectively.

Smad3 is the canonical mediator of TGFβ1 signaling. While we did not identify any Smad binding elements in the −350bp promoter region, several of these were identified upstream of this in the full promoter. We thus assessed the importance of Smad3 to promoter activation by TGFβ1 in MC derived from Smad3 knockout compared with wild-type mice. Western blotting for Smad3 confirmed absence of Smad3 in knockout MC compared with wild-type cells (Figure 5A). Figure 5B shows that activation of the full promoter by TGFβ1 was abolished in the absence of Smad3, identifying a critical role for this transcription factor in regulating TGFβ1-induced *inhba* expression.

Interestingly, Smad3 deletion also prevented TGFβ1-induced activation of the −350bp promoter (Figure 5C), although this region lacks a Smad binding element. This may be through Smad3 regulation of other transcription factors. We next confirmed lack of *inhba* transcript induction by TGFβ1 in Smad3 knockout cells, shown in Figure 5D. Data are presented normalized to their own genotype. Inhibition of Smad3 using SIS3 similarly prevented activation of both the full length and −350bp promoter regions by TGFβ1 (Figures 5E,F). These data highlight an important role for Smad3 in regulation of *inhba* synthesis in response to TGFβ1.

**FIGURE 6:**
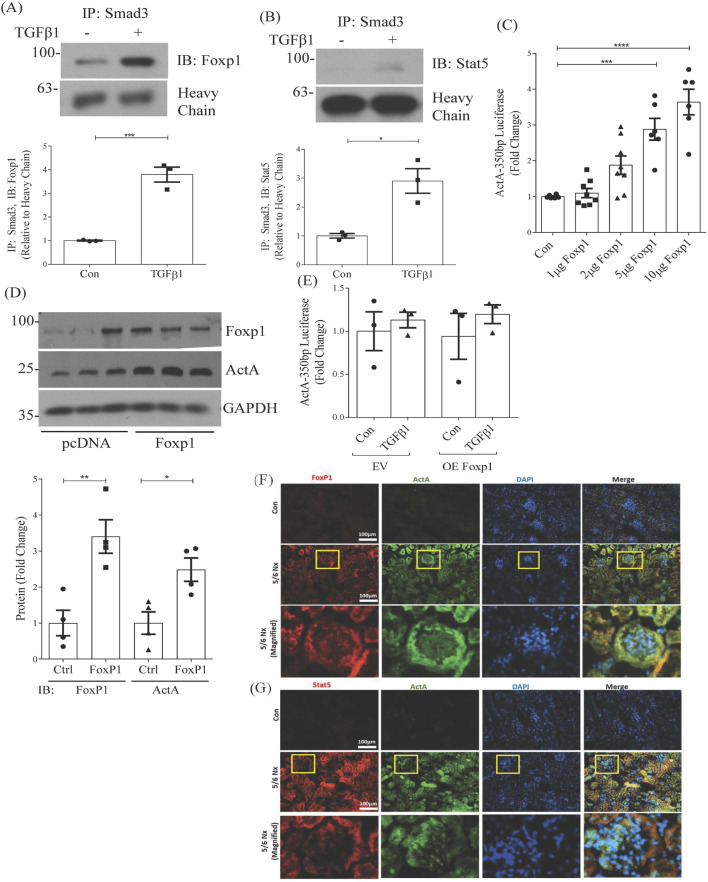
TGFβ1 promotes Foxp1 and Stat5 interaction with Smad3, and their nuclear localization is seen in a CKD model. Immunoprecipitation of Smad3 from MC nuclear fractions shows that TGFβ1 (6 h) increases nuclear localization of **(A)** Foxp1 (n = 3) and **(B)** Stat5 (n = 3). **(C)** Overexpression of FoxP1 dose-dependently increases activation of the −350bp promoter luciferase (n = 7) and **(D)** activin A protein synthesis (n = 4). **(E)** FoxP1 overexpression is unable to increase *inhba* promoter luciferase activation in Smad3 knockout MC (n = 3). **(F,G)** Immunofluorescent staining of kidneys taken 16 weeks after sham and 5/6 nephrectomy in C57BL/6 mice shows increased nuclear localization of **(F)** FoxP1 and **(G)** Stat5, in conjunction with increased expression of activin **(A)**. Boxes identify glomeruli, images for which are magnified for better visualization of glomerular staining. *, **, ***,****P < 0.05, 0.01, 0.001, 0.0001 respectively.

To further investigate the potential interaction of FoxP1 and Stat5 with Smad3, we immunoprecipitated Smad3 from nuclear lysate after treatment with TGFβ1 and probed for FoxP1 or Stat5. Figures 6A,B and show that association of both transcription factors increased significantly in response to TGFβ1. Furthermore, overexpression of FoxP1 in Smad3 wild-type MC dose-dependently increased activity of the −350bp construct (Figure 6C) as well as activin A protein production (Figure 6D), but promoter luciferase activation was completely abrogated in Smad3 knockout cells (Figure 6E). Together, these data suggest that Smad3 interaction with FoxP1 and Stat5 supports their regulation of the *inhba* promoter.

### 3.6 *In vivo* support for FoxP1 and Stat5 regulation of *inhba*

We next sought *in vivo* support for FoxP1 and Stat5 involvement in the upregulation of activin A. We used the well-established 5/6 nephrectomy model of chronic kidney disease to assess whether increased FoxP1 or Stat5 could be colocalized with elevated expression of activin A. We performed immunofluorescence staining of kidneys. In Figures 6F,G, increased colocalization of activin A with FoxP1 or Stat5 is seen after 5/6 nephrectomy. Boxes identify glomeruli which are shown magnified, and suggest presence within areas of MC in addition to the tubular cell staining that is seen. Colocalization using the MC marker integrin α8 is shown in Supplementary Figure S1. These data support the regulation of activin A by these transcription factors *in vivo*.

## 4 Discussion

**FIGURE 7:**
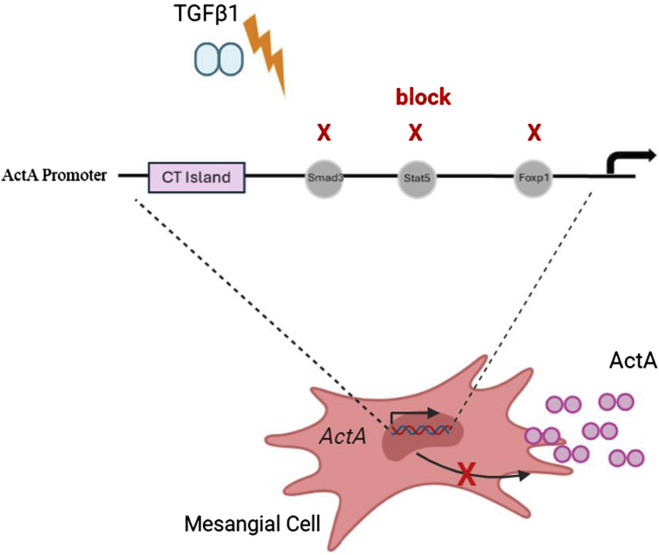
TGFβ1-induced activin A transcriptional upregulation depends on Smad3, Stat5 and Foxp1. Summary schematic illustrating the requirement for transcription factors Smad3, Stat5 and FoxP1 for TGFβ1-stimulated upregulation of *inhba* gene transcription and downstream increased activin A (actA) secretion. A CT island is important for basal transcription, with little activin A expression found in normal kidneys.

Activin A is an important regulator of the profibrotic actions of TGFβ1. While its increased synthesis in response to TGFβ1 is well established, the mechanism by which this occurs remains poorly understood. In this study, we highlight the regulation of *inhba* by TGFβ1 at the transcriptional level in both kidney MC and in chronic kidney disease, and identify the responsive promoter region. We further identify the transcription factors Smad3, FoxP1 and Stat5 as important regulators of *inhba* transcript expression in response to TGFβ1 (Figure 7), and of a CT microsatellite region as a regulator of the basal expression of *inhba*. These data provide an important understanding of the mechanisms regulating activin A production which can inform further activin-targeted therapeutic development.

Our study identifies 350bp upstream of the transcription start site as a critical regulatory region for *inhba* transcription. Similar findings were made by Yoshida et al., with identification of a 400bp region having a strong effect on mouse *inhba* promoter activity (Yoshida et al., 1998). Although not extensively characterized, several signaling mediators and transcription factors which regulate *inhba* expression in a cell and stimulus specific manner have been identified. These include AP-1 family proteins which bind to a variant cAMP response element (CRE) in granulosa cells (Ardekani et al., 1998), and AP-1 and CREB/ATF transcription factors which bind to TRE and CRE binding sites in human fibrosacroma cells (Tanimoto et al., 1996). In activated murine CD4 Th2 cells, the Th2-specific transcription factor c-Maf synergized with NF-AT at binding sites in close proximity to induce gene transcription (Ogawa et al., 2006). However, the specific transcriptional regulation of the activin A gene by TGFβ1 remained poorly understood. Our analysis of regulatory elements in the 350bp TGFβ1 responsive region, supported by mutation studies, identified the transcription factors Stat5 and FoxP1 as key regulators of induction of the activin A gene by TGFβ1.

Stat5 belongs to the Stat family of transcription factors and mediators of cytokine and growth hormone signaling. Stat1 and Stat3 have been the best studied in kidney disease, shown to mediate fibrosis in several models (Chuang and He, 2010). Less is known of the role of Stat5 in kidney fibrosis, but its increase in response to TGFβ1 was shown in MC, and increased Stat5 activation was found in human diabetic kidney disease (Brizzi et al., 2004). Interestingly, Stat5 had antifibrotic effects in a liver model of fibrosis (Hosui et al., 2009). Our data showing an important role for Stat5 in increased activin A production suggest a profibrotic role for Stat5 in the kidney, although this needs further investigation.

FoxP1 is a member of the Forkhead box family of transcription factors which regulate differentiation and function of many tissues. Little is known of the role of FoxP1 in kidney fibrosis. In smooth muscle cells, TGFβ1 was shown to increase FoxP1 expression (Bot et al., 2011). In MC, FoxP1 overexpression reduced oxidative stress and matrix protein production in response to high glucose (Xiang et al., 2019). However, we recently identified that FoxP1 cooperates with transcription factor NFAT5 to upregulate the pan-protease inhibitor alpha 2-macroglobulin, a pathologic regulator of TGFβ1 synthesis, activation and downstream profibrotic signaling (Trink et al., 2024). Interestingly, we note that the binding sites for FoxP1 and Stat5 are in very close proximity in the *inhba* promoter. Future studies would assess whether these two transcription factors synergize in their regulation of *inhba* transcription.

The profibrotic role of Sox9 has been gaining increased recognition. In kidney fibroblasts, Sox9 mediated TGFβ1-induced matrix protein expression, and Sox9 downregulation protected against fibrosis in the unilateral ureteral obstruction model of kidney fibrosis (Li et al., 2018). Sox9 was also shown to increase expression of inhibin βB, the subunit for activin B, in kidney tubular epithelial cells (Sun et al., 2022). Like activin A, activin B was shown to promote kidney fibrosis, but whether the regulation and contribution to fibrosis of these two activins differ is not as yet understood. Here we do not show a role for Sox9 in the regulation of inhibin βA, contrary to what has been shown for inhibin βB. These data suggest differential regulation of these activin genes.

Smad3 regulation of *inhba* transcription was expected given the prominent role of this transcription factor in transmitting TGFβ1 signals. However, the dependence of the 350bp promoter region on Smad3 despite absence of its cognate binding element was unexpected. These findings suggest that Smad3 may interact with and regulate other TGFβ1 signaling mediators that bind to this region of the promoter (Derynck and Budi, 2019). Indeed, our data show that TGFβ1 induced the nuclear interaction of Smad3 with FoxP1 and Stat5, both of which have functional regulatory elements in this region of the promoter. Smad3 interaction with both has also previously been described, albeit in the context of negative gene regulation (Stephen et al., 2014; Das et al., 2022). Our data thus newly suggest a cooperative positive interaction of FoxP1 and Stat5 with Smad3 in mediating *inhba* gene regulation.

Our results indicate an inhibitory effect on promoter transcriptional activation of the microsatellite region between 577 and 437bp containing cytosine (C) and thymine (T) nucleotide repeats. Microsatellites containing CT/AG motifs can form H-DNA triplex structures, impacting gene expression by modifying local DNA structure to either facilitate or impede transcription. CT repeats located in the 5′UTR have been shown to decrease gene expression, eliciting stronger effects with longer tandems. Microsatellites located in the promoter region of a gene also play a role in modulating gene expression by expanding or contracting the length of the initiation site, thus affecting accessibility to the transcriptional machinery. Indeed, 96% of promoter microsatellites associated with gene expression were also found to influence local cytosine methylation status, with increased methylation known to inhibit transcription (Bagshaw, 2017). Our study is the first to identify a CT microsatellite in the *inhba* promoter region. Future studies examining the precise nature of regulation by the *inhba* promoter microsatellite and its influence across different cell types and disease contexts are needed.

In summary, our results identify important regulators of *inhba* transcription. A CT-based microsatellite serves to finetune promoter activity, and Stat5, FoxP1 and Smad3 are required for its induction by TGFβ1. While the importance of TGFβ1 to kidney fibrosis is established, its inhibition is not feasible clinically given its important role in homeostasis (Trionfini and Benigni, 2017). However, the lack of activin A expression in normal kidney, its upregulation in fibrosis, and its importance in mediating TGFβ1 fibrotic effects (Mehta and Krepinsky, 2020; Soomro et al., 2023) make it an attractive therapeutic target. Understanding the nature of its regulation in different diseases may open up avenues for future gene targeting.

## Data Availability

Materials and additional details on methods are available on request. All relevant data is contained within the article except for analysis of the publicly accessible dataset. Information to access this is contained within the article.

## References

[B1] Ardekani A. M. Romanelli J. C. Mayo K. E. (1998). Structure of the rat inhibin and activin betaA-subunit gene and regulation in an ovarian granulosa cell line. Endocrinology 139 (7), 3271–3279. 10.1210/endo.139.7.6116 9645703

[B2] Bagshaw A. T. M. (2017). Functional mechanisms of microsatellite DNA in eukaryotic genomes. Genome Biol. Evol. 9 (9), 2428–2443. 10.1093/gbe/evx164 28957459 PMC5622345

[B3] Bian X. Griffin T. P. Zhu X. Islam M. N. Conley S. M. Eirin A. (2019). Senescence marker activin A is increased in human diabetic kidney disease: association with kidney function and potential implications for therapy. BMJ Open Diabetes Res. Care 7 (1), e000720. 10.1136/bmjdrc-2019-000720 31908790 PMC6936543

[B4] Billings P. C. Bizzaro C. Yang E. Chung J. Mundy C. Pacifici M. (2020). Human and mouse activin genes: divergent expression of activin A protein variants and identification of a novel heparan sulfate-binding domain in activin B. PLoS One 15 (2), e0229254. 10.1371/journal.pone.0229254 32074129 PMC7029874

[B5] Bot P. T. Grundmann S. Goumans M. J. de Kleijn D. Moll F. de Boer O. (2011). Forkhead box protein P1 as a downstream target of transforming growth factor-beta induces collagen synthesis and correlates with a more stable plaque phenotype. Atherosclerosis 218 (1), 33–43. 10.1016/j.atherosclerosis.2011.05.017 21683954

[B6] Brizzi M. F. Dentelli P. Rosso A. Calvi C. Gambino R. Cassader M. (2004). RAGE- and TGF-beta receptor-mediated signals converge on STAT5 and p21waf to control cell-cycle progression of mesangial cells: a possible role in the development and progression of diabetic nephropathy. FASEB J. 18 (11), 1249–1251. 10.1096/fj.03-1053fje 15180953

[B7] Chuang P. Y. He J. C. (2010). JAK/STAT signaling in renal diseases. Kidney Int. 78 (3), 231–234. 10.1038/ki.2010.158 20631733

[B8] Das R. Giri J. P K. P. Froelich N. Chinnadurai R. McCoy S. (2022). A STAT5-Smad3 dyad regulates adipogenic plasticity of visceral adipose mesenchymal stromal cells during chronic inflammation. NPJ Regen. Med. 7 (1), 41. 10.1038/s41536-022-00244-5 36045134 PMC9433418

[B9] Derynck R. Budi E. H. (2019). Specificity, versatility, and control of TGF-beta family signaling. Sci. Signal 12 (570), eaav5183. 10.1126/scisignal.aav5183 30808818 PMC6800142

[B10] Hedger M. P. de Kretser D. M. (2013). The activins and their binding protein, follistatin-Diagnostic and therapeutic targets in inflammatory disease and fibrosis. Cytokine Growth Factor Rev. 24 (3), 285–295. 10.1016/j.cytogfr.2013.03.003 23541927

[B11] Hosui A. Kimura A. Yamaji D. Zhu B. M. Na R. Hennighausen L. (2009). Loss of STAT5 causes liver fibrosis and cancer development through increased TGF-{beta} and STAT3 activation. J. Exp. Med. 206 (4), 819–831. 10.1084/jem.20080003 19332876 PMC2715112

[B12] Kovesdy C. P. (2022). Epidemiology of chronic kidney disease: an update 2022. Kidney Int. Suppl. 12 (1), 7–11. 10.1016/j.kisu.2021.11.003 35529086 PMC9073222

[B13] Li H. Cai H. Deng J. Tu X. Sun Y. Huang Z. (2018). TGF-beta-mediated upregulation of Sox9 in fibroblast promotes renal fibrosis. Biochim. Biophys. Acta Mol. Basis Dis. 1864 (2), 520–532. 10.1016/j.bbadis.2017.11.011 29158184

[B14] Lima F. Mawad H. El-Husseini A. A. Davenport D. L. Malluche H. H. (2019). Serum bone markers in ROD patients across the spectrum of decreases in GFR: activin A increases before all other markers. Clin. Nephrol. 91 (4), 222–230. 10.5414/CN109650 30862350 PMC6595397

[B15] Massague J. Sheppard D. (2023). TGF-beta signaling in health and disease. Cell 186 (19), 4007–4037. 10.1016/j.cell.2023.07.036 37714133 PMC10772989

[B16] Mehta N. Krepinsky J. C. (2020). The emerging role of activins in renal disease. Curr. Opin. Nephrol. Hypertens. 29 (1), 136–144. 10.1097/MNH.0000000000000560 31714286

[B17] Mehta N. Zhang D. Li R. Wang T. Gava A. Parthasarathy P. (2019). Caveolin-1 regulation of Sp1 controls production of the antifibrotic protein follistatin in kidney mesangial cells. Cell Commun. Signal 17 (1), 37. 10.1186/s12964-019-0351-5 30995923 PMC6472091

[B18] Namwanje M. Brown C. W. (2016). Activins and inhibins: roles in development, physiology, and disease. Cold Spring Harb. Perspect. Biol. 8 (7), a021881. 10.1101/cshperspect.a021881 27328872 PMC4930927

[B19] Ogawa K. Funaba M. Chen Y. Tsujimoto M. (2006). Activin A functions as a Th2 cytokine in the promotion of the alternative activation of macrophages. J. Immunol. 177 (10), 6787–6794. 10.4049/jimmunol.177.10.6787 17082592

[B20] Ruiz-Ortega M. Rayego-Mateos S. Lamas S. Ortiz A. Rodrigues-Diez R. R. (2020). Targeting the progression of chronic kidney disease. Nat. Rev. Nephrol. 16 (5), 269–288. 10.1038/s41581-019-0248-y 32060481

[B21] Soomro A. Khajehei M. Li R. O'Neil K. Zhang D. Gao B. (2023). A therapeutic target for CKD: activin A facilitates TGFβ1 profibrotic signaling. Cell Mol. Biol. Lett. 28 (1), 10. 10.1186/s11658-023-00424-1 36717814 PMC9885651

[B22] Stephen T. L. Rutkowski M. R. Allegrezza M. J. Perales-Puchalt A. Tesone A. J. Svoronos N. (2014). Transforming growth factor beta-mediated suppression of antitumor T cells requires FoxP1 transcription factor expression. Immunity 41 (3), 427–439. 10.1016/j.immuni.2014.08.012 25238097 PMC4174366

[B23] Sun Y. Cai H. Ge J. Shao F. Huang Z. Ding Z. (2022). Tubule-derived INHBB promotes interstitial fibroblast activation and renal fibrosis. J. Pathol. 256 (1), 25–37. 10.1002/path.5798 34543458

[B24] Tanimoto K. Yoshida E. Mita S. Nibu Y. Murakami K. Fukamizu A. (1996). Human activin betaA gene. Identification of novel 5' exon, functional promoter, and enhancers. J. Biol. Chem. 271 (51), 32760–32769. 10.1074/jbc.271.51.32760 8955111

[B25] Trink J. Li R. Gao B. Lu C. Krepinsky J. C. (2024). Modulators of alpha-2 macroglobulin upregulation by high glucose in glomerular mesangial cells. Biomolecules 14 (11), 1444. 10.3390/biom14111444 39595620 PMC11592121

[B26] Trionfini P. Benigni A. (2017). MicroRNAs as master regulators of glomerular function in health and disease. J. Am. Soc. Nephrol. 28 (6), 1686–1696. 10.1681/ASN.2016101117 28232619 PMC5461805

[B27] Tsai M. T. Ou S. M. Lee K. H. Lin C. C. Li S. Y. (2023). Circulating activin A, kidney fibrosis, and adverse events. Clin. J. Am. Soc. Nephrol. 19 (2), 169–177. 10.2215/CJN.0000000000000365 37983094 PMC10861103

[B28] Uttarwar L. Gao B. Ingram A. J. Krepinsky J. C. (2012). SREBP-1 activation by glucose mediates TGF-beta upregulation in mesangial cells. Am. J. Physiol. Ren. Physiol. 302 (3), F329–F341. ajprenal.00136.2011 [pii]. 10.1152/ajprenal.00136.2011 22031849

[B29] Xiang H. Xue W. Wu X. Zheng J. Ding C. Li Y. (2019). FOXP1 inhibits high glucose-induced ECM accumulation and oxidative stress in mesangial cells. Chem. Biol. Interact. 313, 108818. 10.1016/j.cbi.2019.108818 31494106

[B30] Yoshida E. Tanimoto K. Murakami K. Fukamizu A. (1998). Isolation and characterization of 5'-regulatory region of mouse activin beta A subunit gene. Biochem. Mol. Biol. Int. 44 (2), 325–332. 10.1080/15216549800201342 9530515

[B31] Zhang X. Liu Y. Fan C. Wang L. Li A. Zhou H. (2017). Lasp1 promotes malignant phenotype of non-small-cell lung cancer via inducing phosphorylation of FAK-AKT pathway. Oncotarget 8 (43), 75102–75113. 10.18632/oncotarget.20527 29088849 PMC5650404

[B32] Zhang D. Gava A. L. Van Krieken R. Mehta N. Li R. Gao B. (2019). The caveolin-1 regulated protein follistatin protects against diabetic kidney disease. Kidney Int. 96 (5), 1134–1149. 10.1016/j.kint.2019.05.032 31492508

