# Supplementary figures and images for: Analysis of TGFβ1-Induced activin A gene expression in kidney mesangial cells
Source: Front Mol Biosci. 2025 Sep 23;12:1607043. doi: 10.3389/fmolb.2025.1607043 (PMC12500454; doi:10.3389/fmolb.2025.1607043)

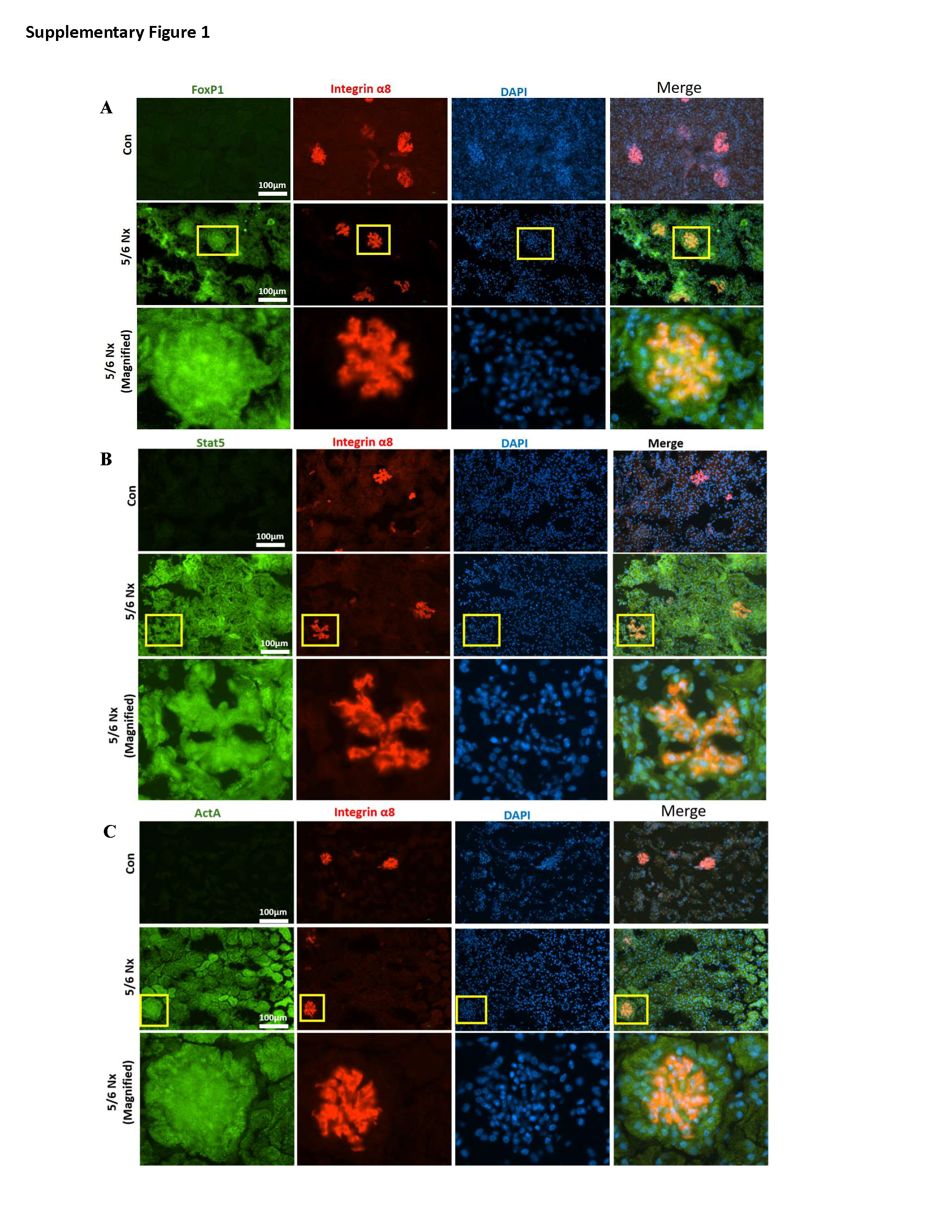

Supplement: Supplementary file 1 [file Image1.tiff]
